# Health Disparities in Achieving Sustained Virologic Response Among Patients With Chronic Hepatitis C: A Retrospective Single-Center Study

**DOI:** 10.7759/cureus.92304

**Published:** 2025-09-14

**Authors:** Eileen Leach, Ricky Rana, Cynthia Nguyen, Jessica Reinecke, Alexander Hall, Manasa Velagapudi

**Affiliations:** 1 Department of Infectious Diseases, Creighton University School of Medicine, Omaha, USA; 2 Department of Infectious Diseases, St. Joseph's Hospital and Medical Center, Creighton University School of Medicine, Phoenix, USA; 3 Department of Clinical Research and Public Health, Creighton University School of Medicine, Omaha, USA

**Keywords:** cirrhosis, direct-acting antivirals, ethnicity, health disparity, hepatitis c virus, race, sustained virologic response

## Abstract

Introduction

Hepatitis C virus (HCV) infection remains a significant public health concern, though direct-acting antivirals (DAAs) have achieved a cure. Sustained virologic response (SVR) at 12 weeks is the most common metric used to assess the efficacy of antiviral therapy in eliminating HCV from the body. Varying treatment outcomes have been reported across different demographic and socioeconomic groups. Understanding the existence and implications of health disparities in HCV treatment is crucial for designing equitable healthcare interventions. In this study, we aimed to describe the potential health disparities related to achieving SVR.

Methods

A retrospective chart review was conducted of adult (19+ years) patients treated for HCV in Gastroenterology and Infectious Diseases clinics at a single institution from 2018 to 2022. Log binomial regression was used to estimate the adjusted likelihood of achieving SVR; covariates included age, sex, race, marital status, median household income by zip code, presence of cirrhosis on imaging, and the DAA used for treatment.

Results

Among the study population, 285 (79%) patients achieved SVR. Although there were no statistically significant differences in the likelihood of achieving SVR observed across any covariate, several factors approached significance. Asian patients were less likely to achieve SVR compared to Caucasian patients, with a relative risk (RR) of 0.92 (95% confidence interval (CI), 0.80-1.05). Cirrhotic patients were more likely to achieve SVR compared to non-cirrhotic patients with a RR of 1.15 (0.99-1.32). Married patients were more likely to achieve SVR compared to single or widowed patients, with a RR of 1.12 (0.98-1.27).

Conclusion

We observed a higher likelihood of achieving SVR in Caucasian patients, cirrhotic patients, and married patients, although none of the differences achieved statistical significance. Further studies with larger sample sizes are needed to elucidate the disparities and better guide targeted measures to improve them.

## Introduction

Hepatitis C virus (HCV) infection is a major public health concern, with an estimated 51 million people infected worldwide and 2.5 million in the United States [1]. Chronic hepatitis C causes continued inflammation in the liver, which may progress to cirrhosis. Patients with cirrhosis are at increased risk of liver failure, hepatocellular carcinoma (HCC), and death [2]. Cure is quantified by sustained virologic response (SVR), meaning the HCV is no longer detectable in the blood after treatment completion. Historically, treatment for HCV consisted of dual therapy with pegylated interferon and ribavirin. However, these therapies had substantial side effects and limited virological efficacy, especially in racial minority groups and in the setting of advanced liver disease. Generally, racial disparities in SVR rates have decreased since the advent of direct-acting antivirals (DAAs), with SVR rates across racial groups typically exceeding 90% [3]. However, there are still patients who are unresponsive to treatment. This could potentially be attributed to viral resistance, but previous studies have demonstrated that health disparities, such as race and socioeconomic status, may play a crucial role in limiting access to DAAs [4]. The disparities present in HCV treatment must therefore be researched and addressed to better treat patients. Previous studies have demonstrated similar rates of SVR regardless of race, age, socioeconomic status, choice of DAA, and presence of cirrhosis [5-8]. 

The primary aim of our single-center retrospective study was to determine if demographic factors, including race, sex, marital status, age, and presence of cirrhosis, impacted the likelihood of achieving SVR. Further studies on SVR rates will allow healthcare professionals to better serve a diverse population.

## Materials and methods

We performed a retrospective chart review of adults aged 19 years and older who were treated with DAA-only regimens (sofosbuvir/velpatasvir, glecaprevir/pibrentasvir, ledipasvir/sofosbuvir, or other DAA regimen), without the addition of ribavirin or interferon. Patients were treated at gastroenterology and infectious disease clinics affiliated with a single institution in Omaha, Nebraska, a large Midwestern city, between 2018 and 2022 (Table 1). The measured outcome was SVR at 12 weeks. We included patients who were at least 19 years of age, had a confirmed diagnosis of chronic HCV infection, and initiated treatment with DAAs between 2018 and 2022 at our institution. Patients were excluded if they were less than 19 years of age, had no documented HCV infection, did not initiate treatment, were lost to follow-up prior to SVR assessment, or had incomplete treatment records. The age of majority in Nebraska is 19 years, so we used this definition of adulthood in our analysis. Baseline characteristics, including sex, age, and marital status, were recorded for each qualifying patient, along with the prescribed DAA regimen. Race and ethnicity data were collected from the electronic medical record (EMR), where patients self-reported using the available categories: “Caucasian,” “Asian,” “Hispanic,” and “Other.” More granular racial or ethnic categories were not available within the EMR system and, therefore, were not captured in our analysis. If patients had cirrhosis on ultrasound or computed tomography at the time of diagnosis, this was also recorded. Treatment duration was determined by the specific DAA regimen used and by the presence or absence of cirrhosis, consistent with guideline-based practice. Household income for each individual patient was not known but was estimated based on U.S. Census Bureau data per reported patient zip code [9]. Log binomial regression was used to estimate the adjusted likelihood of achieving SVR. Covariates included age, sex, race, marital status, median household income by zip code, presence of cirrhosis on imaging, and the DAAs used for treatment. Ethnicity was considered but not included in adjusted models due to insufficient sample size. All analyses were conducted using Statistical Analysis Software (SAS) v. 9.4 (SAS Institute, Cary, NC). Statistical significance was determined using a two-tailed p<0.05.

**Table 1:** Patient characteristics presented as number of patients (n) and percentage of total population (%) for each criterion The data have been descriptively presented as the number of patients and the percentage of the total patient population meeting each criterion, or as the median and interquartile range. N: number of patients; IQR: interquartile range; SVR: sustained virologic response; DAA: direct-acting antiviral

N=363	N (%)
Gender	
Female	140 (39%)
Male	223 (61%)
Outcome	
Cirrhosis	68 (19%)
No cirrhosis	295 (81%)
Marital status	
Single/widower	184 (51%)
Married	120 (33%)
Divorced/ legally separated	59 (16%)
DAA	
Sofosbuvir/velpatasvir	165 (45%)
Ledipasvir/Sofosbuvir	55 (15%)
Glecaprevir/pibrentasvir	127 (35%)
Other	16 (4%)
Race	
Caucasian	223 (61%)
Asian	110 (30%)
Other	30 (8%)
Ethnicity	
Hispanic	7 (2%)
Achieved SVR	285 (79%)
Age-median (IQR)	60 (53-66)
Median household income-median (IQR)	$57980 (49630-72143)

This study was conducted in compliance with the Declaration of Helsinki and was approved by the Institutional Review Board (IRB) of the Creighton University School of Medicine for research involving human subjects (protocol number 2003446-01; application date: November 14, 2022).

## Results

Over the study period, 363 patients were treated, with 285 (79%) achieving SVR. Patients were treated with sofosbuvir/velpatasvir (n=165, 45%), glecaprevir/pibrentasvir (n=127, 35%), ledipasvir/sofosbuvir (n=55, 15%), or other DAAs (n=16, 4%) (Table 1). There were no statistically significant differences in the likelihood of achieving SVR observed across any covariate (Table 2). The majority of patients were Caucasian (n=223, 61%) or Asian (n=110, 30%), with 30 (8%) belonging to other races. Because there were few patients from racial groups besides Caucasian and Asian, they were classed as “other” for the purposes of statistical analysis. Asian patients had an 8% lower likelihood of achieving SVR compared to Caucasian patients, with an adjusted relative risk (RR) of 0.92 (0.80-1.05). There were more male patients (n=223, 61%) than female patients (n=140, 39%) treated in our study, though males and females were not found to have a significant difference in likelihood of achieving SVR with an adjusted RR of 1.05 (95% confidence interval (CI), 0.91-1.21). Median patient age was 60 years, though patient age was not associated with achievement of SVR with an adjusted RR 1.01 (95% CI, 0.99-1.03). Median household income was also not associated with a significant difference in achieving SVR with an adjusted RR of 0.99 (95% CI, 0.97-1.01). The majority of patients (n=295, 81%) did not have cirrhosis, and cirrhosis was not associated with a significant difference in rate of SVR with an adjusted RR of 1.15 (95% CI, 0.99-1.32), though this factor did approach significance. Patients were more commonly single or widowed (n=184, 51%) than married (n=120, 33%) or divorced/legally separated (n=59, 16%). Marital status, however, was not significantly associated with a difference in rates of SVR.

**Table 2:** Characteristics of patients achieving SVR, unadjusted and adjusted risk ratios with p<0.05 The data have been presented as both unadjusted and adjusted risk ratios, with adjustments made for all reported variables. Statistical significance was determined using a two-tailed test with p<0.05. N: number of patients; RR: risk ratio; CI: confidence interval; DAA: direct-acting antiviral; SVR: sustained virologic response

N=363	Unadjusted RR (95% CI)	Adjusted RR (95% CI)	| t |	P_Adj_
Age (5 years)	1.01 (0.99-1.03)	1.01 (0.99-1.03)	1.07	0.283
Median household income	0.99 (0.98-1.00)	0.99 (0.97-1.01)	1.67	0.096
Female vs male	0.96 (0.85-1.07)	1.05 (0.91-1.21)	0.68	0.497
Cirrhosis vs no	1.16 (1.04-1.29)	1.15 (0.99-1.32)	1.88	0.061
Marital status				
Married vs single/widower	1.05 (0.93-1.18)	1.12 (0.98-1.27)	1.66	0.097
Divorced/separated vs single/widower	1.01 (0.86-1.18)	0.99 (0.83-1.19)	0.06	0.952
Divorced/separated vs married	0.96 (0.82-1.13)	0.89 (0.73-1.08)	1.16	0.246
DAA				
Ledipasvir/sofosbuvir vs sofosbuvir/velpatasvir	1.06 (0.92-1.22)	1.09 (0.93-1.28)	1.09	0.277
Glecaprevir/pibrentasvir vs sofosbuvir/velpatasvir	0.95 (0.83-1.08)	0.97 (0.85-1.10)	0.51	0.610
Ledipasvir/sofosbuvir vs glecaprevir/pibrentasvir	1.12 (0.96-1.31)	1.13 (0.94-1.34)	1.38	0.169
Other vs sofosbuvir/velpatasvir	1.11 (0.91-1.36)	1.17 (0.91-1.50)	1.21	0.228
Other vs ledipasvir/sofosbuvir	1.05 (0.84-1.30)	1.07 (0.81-1.40)	0.48	0.634
Other vs glecaprevir/pibrentasvir	1.17 (0.95-1.45	1.21 (0.93-1.58)	1.40	0.163
Race				
Asian vs Caucasian	1.01 (0.90-1.14)	0.92 (0.80-1.05)	1.25	0.211
Other vs Caucasian	1.03 (0.85-1.24)	1.03 (0.84-1.26)	0.27	0.790
Other vs Asian	1.01 (0.83-1.24)	1.12 (0.90-1.40)	1.00	0.316

## Discussion

Although a variety of HCV antivirals are now available, some patients remain unresponsive to treatment. While viral resistance is often a contributing factor, we sought to identify potential health disparities as additional determinants of poor outcomes, because targeted interventions could help mitigate these issues [10].

In our retrospective review of HCV patients treated with DAAs, we did not find any baseline characteristic, such as age, median income, gender, presence of cirrhosis, marital status, choice of DAA, or race, that was statistically associated with a lower likelihood of achieving SVR when controlling for covariates. Prior studies have also shown no association between rates of SVR and cirrhosis or DAA [5,6]. Cirrhotic patients typically respond to DAAs and achieve SVR [5]. In our study, cirrhotic patients were more likely to achieve SVR than non-cirrhotic patients, though this difference was non-significant. We hypothesize that cirrhotic patients might have been more motivated to adhere to treatment due to the severity of their condition. Cirrhotic patients are notably at higher risk for adverse events of DAAs, such as transaminitis, but our study did not assess adverse events of treatment [5]. Rates of SVR across DAAs typically exceed 90 [6]. In our analysis, we found that age and sex were not significantly associated with achievement of SVR. Prior studies have shown that women were more likely to achieve SVR than men. Though the mechanism remains unclear, this could potentially be due to better treatment adherence among women [11,12]. Because we excluded patients who did not adhere to treatment, this might explain why we did not find a difference by sex.

Our findings were consistent with previous studies that showed SVR rates were similar across race [7,8]. In their cohort study in California, Le et al. demonstrated that although there was no difference in SVR between Caucasian and Asian or Hispanic patients, Asian and Hispanic patients who did not achieve SVR had a greater likelihood of developing cirrhosis or HCC [8]. Their study highlights the importance of achieving SVR, particularly in racial minorities, who have a higher incidence of adverse outcomes if they do not achieve SVR. Bryant et al. demonstrated that among safety net hospitals, rates of SVR were not significantly different across racial groups but were decreased by patients being lost to follow-up [7]. This once again highlights the importance of patient adherence as a barrier to treatment. Our results suggest that Asian patients had an approximately 8% lower likelihood of achieving SVR compared to Caucasian patients; however, this difference was not statistically significant. Asian Americans have been previously shown to have a different distribution of HCV genotypes than other ethnic groups [8,13]. Conversely, Lim et al. reported that despite carrying genotypes typically associated with poorer treatment response in Caucasian populations, Asians achieved high rates of SVR [14]. We hypothesize that the Asian patients in our study might have had genotypic variations that responded poorly to the chosen DAA, leading to slightly lower rates of SVR. While genotype distribution may contribute to outcome differences, we could not include this in adjusted models as genotype data were not consistently available across our retrospective dataset. The lack of consistently available genotyping was likely because currently available DAA regimens are pan-genotypic and highly effective across all HCV genotypes [13]. This has reduced the routine clinical use of genotype testing. However, we also acknowledge that genotype differences could still have influenced SVR rates, and the absence of this information represents a limitation of our study. This could be an area of focus for future research.

Though we could not directly account for the socioeconomic status of each patient, we used the mean income per patient zip code reported by the U.S. Census Bureau as an estimate of income. We found no significant association between median household income and achievement of SVR. Overall, the patients in our study had an estimated median household income of $57980. This was lower than the overall median income of $71238 in Omaha, Nebraska. However, 13.4% of Omaha’s residents live below the poverty line, and 8.0% do not have health insurance, suggesting that the clinics in our study were likely treating patients from lower socioeconomic backgrounds, which could have potentially contributed to our lower rates of SVR [9]. Although marital status was not significantly associated with rates of SVR, the comparison of SVRs among married and single or widowed patients did approach significance. As household income is affected by the number of people in the household, single or widowed patients likely had a lower income than married patients, which could have contributed to lower rates of SVR.

Our study had several strengths, including its attempt to account for a wide range of potential health disparities and its unique focus on HCV. Prior studies of numerous health conditions have shown associations between factors, including race, socioeconomic status, marital status, and sex, and outcomes.

Our study was limited by a small sample size, among other factors, such as not accounting for the different genotypes of HCV infection. As this was a retrospective study, we did not have complete access to patient data. Patient race was determined based on how they self-classified in the EMR, which did not capture all specific racial groups. The majority of the study patients were Caucasian, and sample sizes from racial groups other than Asians and Caucasians were insufficient to be analyzed by individual group. Future research should prioritize more inclusive and detailed reporting of race and ethnicity to improve equity and precision in health disparities research. We also did not account for HCV genotype or cirrhosis staging in our analysis. Though cirrhosis staging is clinically relevant and can influence SVR, detailed genotype information and standardized cirrhosis staging (e.g., METAVIR or Child-Pugh scores) were not consistently available across the study period and thus could not be incorporated into the adjusted models [14]. Additionally, we were unable to completely account for potential confounding factors, such as treatment adherence, baseline viral load, or comorbidities. We also did not know specific household incomes for patients in the study, nor could we assess other potential barriers to treatment, such as knowledge of treatment options, transportation availability, and insurance coverage. These limitations might have introduced residual confounding into our study.

## Conclusions

Our study did not find significant differences in rates of SVR associated with race or income. However, our findings are consistent with previous research demonstrating that high SVR rates can be achieved regardless of race, gender, age, income, or the presence of cirrhosis. This consistency is promising for future reductions in the global burden of HCV. More research is needed to understand the impact of disparities on the diagnosis and treatment of HCV and other infectious diseases. Further studies with larger sample sizes are needed to determine whether factors such as race and income are associated with poorer treatment outcomes and to guide targeted measures to improve disparities. Additionally, future studies should incorporate genotype and cirrhosis stage data, as these could help explain the trends observed in Asian patients and further clarify the role of underlying liver disease severity in SVR outcomes. If disparities are identified, providers will have the opportunity to implement interventions to address these gaps in care.
